# Minoxidil Skin Delivery from Nanoemulsion Formulations Containing Eucalyptol or Oleic Acid: Enhanced Diffusivity and Follicular Targeting

**DOI:** 10.3390/pharmaceutics10010019

**Published:** 2018-01-25

**Authors:** Eman Abd, Heather A. E. Benson, Michael S. Roberts, Jeffrey E. Grice

**Affiliations:** 1 Therapeutics Research Centre, School of Medicine, Translational Research Institute, University of Queensland, Brisbane 4102, Australia; e.abd@uq.edu.au (E.A.); m.roberts@uq.edu.au (M.S.R.); 2 Curtin Health Innovation Research Institute, School of Pharmacy, Curtin University, Perth 6845, Australia; H.Benson@curtin.edu.au; 3 School of Pharmacy and Medical Sciences, University of South Australia, Adelaide 5000, Australia

**Keywords:** nanoemulsion, stratum corneum, hair follicles, skin maximum flux, skin penetration enhancers, oleic acid, eucalyptol

## Abstract

In this work, we examined enhanced skin delivery of minoxidil applied in nanoemulsions incorporating skin penetration enhancers. Aliquots of fully characterized oil-in-water nanoemulsions (1 mL), containing minoxidil (2%) and the skin penetration enhancer oleic acid or eucalyptol as oil phases, were applied to full-thickness excised human skin in Franz diffusion cells, while aqueous solutions (1 mL) containing minoxidil were used as controls. Minoxidil in the stratum corneum (SC), hair follicles, deeper skin layers, and flux through the skin over 24 h was determined, as well as minoxidil solubility in the formulations and in the SC. The nanoemulsions significantly enhanced the permeation of minoxidil through skin compared with control solutions. The eucalyptol formulations (NE) promoted minoxidil retention in the SC and deeper skin layers more than did the oleic acid formulations, while the oleic acid formulations (NO) gave the greatest hair follicle penetration. Minoxidil maximum flux enhancement was associated with increases in both minoxidil SC solubility and skin diffusivity in both nanoemulsion systems. The mechanism of enhancement appeared to be driven largely by increased diffusivity, rather than increased partitioning into the stratum corneum, supporting the concept of enhanced fluidity and disruption of stratum corneum lipids.

## 1. Introduction

The horny layer on the skin surface, the stratum corneum (SC), forms a significant barrier that limits the absorption of substances that come in contact with it. However, despite the resistance imposed by the SC, transcutaneous delivery of active substances for local or systemic effects continues to attract interest. One strategy that is employed to reduce the impact of the SC barrier and enhance skin absorption is the use of optimized colloidal formulations such as emulsions. Microemulsions and nanoemulsions are commonly used in topical formulations applied to the skin as therapeutic, cosmeceutical, cosmetic, and personal care products [1]. They offer high solubilization of actives and the potential to manage delivery rates to the skin, in elegant products with good sensorial characteristics. Micro- and nanoemulsions are thermodynamically stable, isotropic systems containing water, oil, surfactants, and cosurfactants in specific ratios. They typically have droplets in the sub 100 micron range with low polydispersity (<10%), and can be designed to encapsulate and enhance the skin delivery of a wide range of hydrophilic and lipophilic drugs [1]. The specific properties of micro- and nanoemulsions, including their low interfacial tension and small droplet size, can be controlled with an appropriate mix of primary surfactant, such as a nonionic surfactant or lecithin; cosurfactant, such as short or medium chain alcohols or polyglyceryl derivatives; and a high surfactant to oil ratio.

By using a combination of suitable excipients, penetration enhancement can be brought about through different mechanisms. Commonly used oil phase components include fatty acids (e.g., oleic acid), esters of fatty acids and alcohols (e.g., isopropyl myristate, isopropyl palmitate, ethyl oleate), medium chain triglycerides, triacetin, and terpenes (e.g., limonene, menthol, cineole), many of which have been shown to enhance percutaneous permeation with minimal irritation to the skin [2,3]. We chose eucalyptol for this work because it has been used previously as a penetration enhancer in topical formulations with minimal skin irritation [4], although we note that adverse effects have been reported. Vilaplana and Romaguera [5] reported a single case of contact dermatitis due to eucalyptol in an anti-inflammatory cream, but pointed out that they had found only one previous reference to a similar response, caused by eucalyptol derived from tea tree oil [6]. One way in which these penetration enhancers affect skin is by disrupting the organization of SC lipids and increasing their fluidity. This has the effect of decreasing the diffusional resistance to compounds [2,7,8]. Surfactants and cosurfactants are also capable of performing this function, and may also increase drug solubility within the stratum corneum. 

Micro- and nanoemulsions may also promote skin absorption due to fluctuations in their oil–water interface affecting the movement of the solute between their lipophilic and hydrophilic domains and into the SC [9], increasing the solubilization capacity for both hydrophilic and lipophilic solutes, and maintaining a constant supply of the solute in the external phase. As well as an ability to enhance skin absorption, micro- and nanoemulsions may also act to reduce the irritant properties of certain excipients. Thus, micro- and nanoemulsions may be regarded as superior vehicles for skin delivery compared to conventional emulsions or gels, due to the interaction of their individual components with the skin and their phase structure and particle size.

Under certain conditions, substances may enter the skin through appendages (hair follicles and possibly sweat glands). The follicular infundibulum of the hair follicle provides an increased surface area with an ever-decreasing SC barrier thickness with depth [10]. The dense network of blood capillaries surrounding the hair follicles may also offer a mechanism for enhanced systemic absorption for substances that penetrate into the follicles [11]. Consequently, hair follicles have potential as a site for delivery of drugs into the viable skin layers, and for targeted delivery to treat conditions related to the hair follicles such as acne and alopecia. The follicular penetration of dyes [12] and nanoparticles [13] has been well studied, but relatively less has been done to examine follicular delivery of therapeutic compounds [14]. Given the presence of sebum in hair follicles, it is likely that the composition and physical properties of these formulations would facilitate transfollicular transport. This makes nano- and microemulsion formulations an attractive option for targeted delivery to the hair follicles for conditions such as acne and alopecia. Increased drug penetration of the lipophilic compounds adapalene [15] and curcumin [16] into hair follicles from microemulsion formulations has been demonstrated. Teichmann et al. showed that, in addition to increased deposition, the microemulsion provided curcumin transport throughout the follicular infundibula, whereas with a coarse emulsion/cream, curcumin delivery was limited to the follicular orifices.

This study aimed to determine the skin delivery and distribution of minoxidil (log *P* 1.24) applied to human skin in oil-in-water (o/w) nanoemulsion formulations containing chemical penetration enhancers with potentially different enhancement mechanisms (oleic acid and eucalyptol). The determination of parameters such as skin permeation flux, diffusivity, partition coefficients, and stratum corneum solubility provided insight into the mechanisms of permeation enhancement occurring with these formulations. The formulations used here were previously developed and used to enhance the permeation of naproxen and caffeine through excised human skin [17]. This work expands those previous studies by examining minoxidil delivery into hair follicles. Differential tape stripping followed by cyanoacrylate skin surface biopsy was performed, together with quantification of minoxidil in skin layers, to determine the extent to which the skin and hair follicles could be targeted.

## 2. Materials and Methods

### 2.1. Chemicals

Minoxidil, ethanol, oleic acid (OA), and eucalyptol (EU) were purchased from Sigma-Aldrich Pty. Ltd. (Sydney, Australia). Volpo-N10 was obtained from Uniqema (Witton Centre, Witton Redcar, UK). All chromatography reagents were analytical reagent grade.

### 2.2. Formulation Preparation and Characterization

The emulsion formulations containing surfactant, co-surfactant, and eucalyptol or oleic acid were developed and characterized as previously described [17]. A ternary phase diagram was constructed using the water titration technique to define the concentration ranges of components that produced clear emulsions. Volpo-N10 (surfactant) was dissolved in ethanol (co-surfactant) in a 1:1 ratio, then mixed with oleic acid or eucalyptol (oil phase) and phosphate buffered saline pH 7.4 (PBS: aqueous phase) in a 0.6:1:1 ratio, followed by gentle mixing. The resulting nanoemulsions were clear at room temperature. Minoxidil (2% *w*/*w*) was dissolved in the nanoemulsion and control solutions. This concentration is used in commercial minoxidil products and was also used in previous skin permeation studies from our laboratory [18]. The compositions of emulsions and control solutions are shown in Table 1. The eucalyptol and oleic acid concentrations used in the emulsion formulations were within the clear emulsion range (approximately 12–22%) shown in the previous study [17]. The PEG-6000/water control solutions were used because these are inert vehicles that do not alter the skin membrane properties and consequently cause no penetration enhancement.

The droplet size distribution, refractive index, electrical conductivity, and viscosity of the emulsions were determined at ambient temperature as previously described [17] and are shown in Table 2.

### 2.3. Human Skin Preparation

Four skin samples were obtained with informed consent from female patients undergoing elective abdominoplasty, and with approval from the University of Queensland Human Research Ethics Committee (HREC Approval no. 2008001342, 25 October 2016). The procedures were conducted in compliance with the guidelines of the National Health and Medical Research Council of Australia. Full thickness skin was prepared by removal of subcutaneous fat by blunt dissection. Epidermal membranes were prepared from full-thickness skin by immersion in water at 60 °C for 1 min, to allow the epidermis to be teased away from the dermis [19]. Stratum corneum (SC) was prepared from the epidermal membranes by trypsin digestion [20]. The epidermis was floated overnight on a solution of 0.01% trypsin in phosphate buffered saline at 37 °C. The digested viable epidermis was gently scraped off with cotton buds and the remaining stratum corneum membrane was rinsed several times with distilled water. The isolated stratum corneum membranes were dried with absorbent paper, placed flat between parafilm sheets and covered with aluminum foil. All skin membranes were stored frozen at −20 °C and used within one month.

### 2.4. Determination of Minoxidil Solubility in the Formulations (S_f_) and in the Stratum Corneum (S_SC_)

The solubility of minoxidil in the formulation (*S_f_*) was determined by adding minoxidil to 5 mL of each nanoemulsion or control solution until an excess amount remained. The samples were incubated in a water bath at 32 °C for 24 h with continuous agitation, then centrifuged at 4700 rpm for 10 min. The supernatant was withdrawn and diluted to accurately quantify the minoxidil content by HPLC (Shimadzu, Tokyo, Japan).

To determine the SC solubility of minoxidil from the various vehicles, preweighed discs of SC (four replicates for each vehicle) were incubated in 1 mL saturated solutions of each vehicle at 32 °C for 24 h [20]. The SC discs were then removed and blotted dry before being incubated with 1 mL of 70% ethanol/water for 24 h at 32 °C to enable complete extraction of the solutes. *S_SC_* was determined from the amount recovered in the extraction fluid measured by HPLC divided by the thickness and area of the SC [21].

### 2.5. In Vitro Skin Permeation Study

In vitro skin permeation studies were performed with full-thickness skin in Franz diffusion cells with an effective diffusion area of 1.33 cm^2^ and approximately 3.4 mL receptor chamber capacity. The skin was cut into discs and mounted between the donor and receptor compartment of the Franz cell with the stratum corneum side facing the donor chamber. The receptor compartment containing PBS (pH 7.4) was immersed in a water bath at 35 ± 0.5 °C. The donor solution consisted of 1 mL of the nanoemulsion or control formulations, containing 2% *w*/*w* minoxidil (except C1, which was a saturated solution). The donor compartment was covered with parafilm to prevent evaporation. At predetermined time points, 200 µL of the receptor phase was withdrawn and replaced with an equal volume of fresh PBS. The minoxidil content in all samples was determined by HPLC.

After Franz diffusion studies were completed, the skin in each cell was wiped with a cotton bud to remove any remaining formulation and then subjected to tape stripping (20 times) by application of adhesive tape (D-Squame tapes, CuDerm Corp., Dallas, TX, USA) to the exposed skin surface for about 5 s, followed by careful removal. The tapes were placed into separate vials (groups of five) and soaked overnight with 2 mL methanol before analysis by HPLC. The amount of absorbed minoxidil in the tapes represented the amount of minoxidil deposited within the SC.

The technique of follicular casting, developed and validated by Lademann’s group [22] and applied previously in our laboratory to quantify follicular delivery of minoxidil [18], was used here. Following tape stripping, one drop of superglue (Loctite; Henkel, Düsseldorf, Germany) was placed on a microscope slide, then pressed onto the surface of the stripped skin with light pressure for 15 min. The slide was then peeled carefully from the skin. The superglue was dissolved by rubbing with acetone-soaked cotton buds (four times), and the cotton buds soaked overnight in 2 mL methanol prior to analysis by HPLC.

After follicular casting, the skin was chopped into small pieces, homogenized, and soaked overnight with 2 mL methanol under constant shaking and at room temperature before analysis by HPLC.

### 2.6. HPLC Analysis of Minoxidil

Minoxidil in receptor fluid, SC, hair follicles, and skin layers was analyzed using a validated HPLC assay. The HPLC system consisted of a Shimadzu SIL-20 a HT, a CBM-20A system controller, an SPD-20A detector, an LC-20AD pump, and an auto injector. Isocratic separation of minoxidil was performed on a Phenomenex Luna C18 5 μ (150 × 4.6 mm) column at ambient temperature with a mobile phase of 60% of 0.01 M sodium dihydrogen phosphate (Na_2_H_2_PO_4_) buffer and 40% acetonitrile *v*/*v*, containing 2.5 mM sodium dodecyl sulphate (SDS) and adjusted to pH 3 (1 mL/min flow rate). The detection wavelength of minoxidil and its internal standard (propyl paraben) is 281 nm.

### 2.7. Data Analysis

The cumulative amount (*Q*, µg/cm^2^) of minoxidil penetrating through an area of 1.3 cm^2^ was plotted against time (*t*). Steady-state flux *J_SS_* (µg/cm^2^/h) was determined from the slope of the linear portion of the cumulative amount (*Q*) versus time plot.

The maximum flux (*J_max_*) that would be applicable to saturated solutions can be estimated from the experimental steady state flux, corrected for the known solubility in the formulation, by Equation (1) [21]:

*J_max_ = J_SS_ S_f_/C_f_* (1)

where *S_f_* is the solubility in the formulation and *C_f_* is the experimental concentration used.

The apparent diffusivity of minoxidil in the skin divided by path length (D*) was calculated from the maximum flux and the solubility of minoxidil in the SC, according to Equation (2) [21]:

D* = J_max_/S_SC_ (2)

where *S_SC_* is the experimentally determined solubility of minoxidil in the stratum corneum. 

### 2.8. Statistical Analysis

All experiments were analyzed by one-way analysis of variance (ANOVA) with post-hoc comparisons (Tukey) using GraphPad Prism 6 (GraphPad Software Inc., La Jolla, CA, USA); *p* < 0.05 was considered to be significant.

Comparisons were made between the nanoemulsion formulations and controls, as well as between the different nanoemulsion formulations, for the cumulative amount permeated at different time points and the amount of minoxidil recovered following tape stripes, follicular casting, and skin extraction.

## 3. Results

### 3.1. In Vitro Permeation of Minoxidil across Full-Thickness Skin

**Figure 1:**
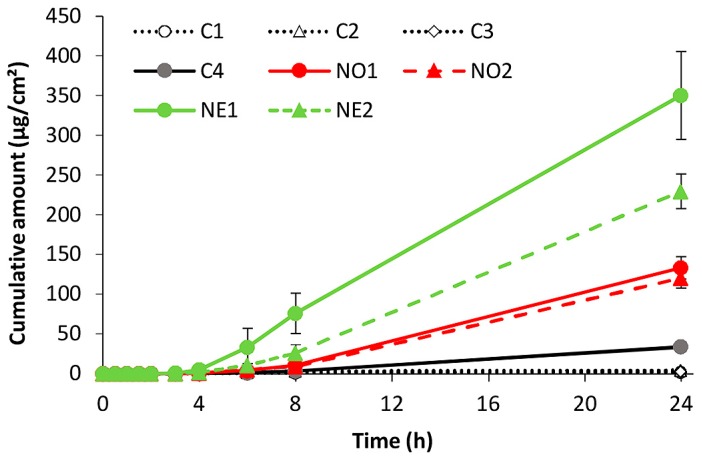
Cumulative amount of minoxidil permeating through full-thickness human skin from nanoemulsions and control vehicles (mean ± SD, *n* = 4).

The cumulative amounts (μg/cm^2^) of minoxidil permeated through full-thickness skin from each nanoemulsion and control are shown in Figure 1. Minoxidil permeation from nanoemulsions was greater than from control solutions. Minoxidil flux (*Jss*) over 24 h was in the order NE1 > NE2 > NO1 ≥ NO2 > C4 > C1 = C2 = C3. The greatest flux was seen with formulations containing eucalyptol as the oil phase, although all nanoemulsion formulations gave significantly greater fluxes than controls. The 60% ethanol solution also showed enhanced minoxidil permeation compared with other controls.

### 3.2. Effect of Formulation on Minoxidil Solubility in Formulation, Solubility in SC, Maximum Flux, and Derived Diffusivity

Estimated minoxidil solubility in formulation, solubility in SC, maximum flux calculated from steady state flux using Equation (1) and diffusivity per path length D* derived from the maximum flux and SC solubility using Equation (2), are shown in Table 3. Minoxidil solubility ranged from 15.5 ± 2.3 to 283.4 ± 2.3 mg/mL and was greater in the nanoemulsion formulations containing enhancers than in controls, in the following rank order: NE2 > NE1 > NO2 > NO1 > C3 > C2 > C4 > C1. The solubility of minoxidil in the SC (*Ssc*) followed a similar pattern, although it was noted that, in the controls, the ethanol/water solution (C4) showed the greatest solubility in the SC. Thus, the data suggests that the nanoemulsions provide only a modest increase in minoxidil solubility in the SC, particularly in comparison to the ethanol/water vehicle.

The maximum flux of minoxidil (*J_max_*) for nanoemulsions (42.6 ± 12.9 to 188.4 ± 23.4 µg/cm^2^/h) was substantially greater compared with all controls (0.4 ± 0.1 to 3.3 ± 0.2 µg/cm^2^/h). The diffusivity D* of minoxidil was also one to two orders of magnitude greater from the nanoemulsions than from the controls (Table 3). The increase in *J_max_* and D*, with only a modest increase in *S_sc_*, suggests that the mechanism of enhanced minoxidil penetration is primarily due to the reduction in SC barrier resistance due to the oleic acid and eucalyptol.

**Figure 2:**
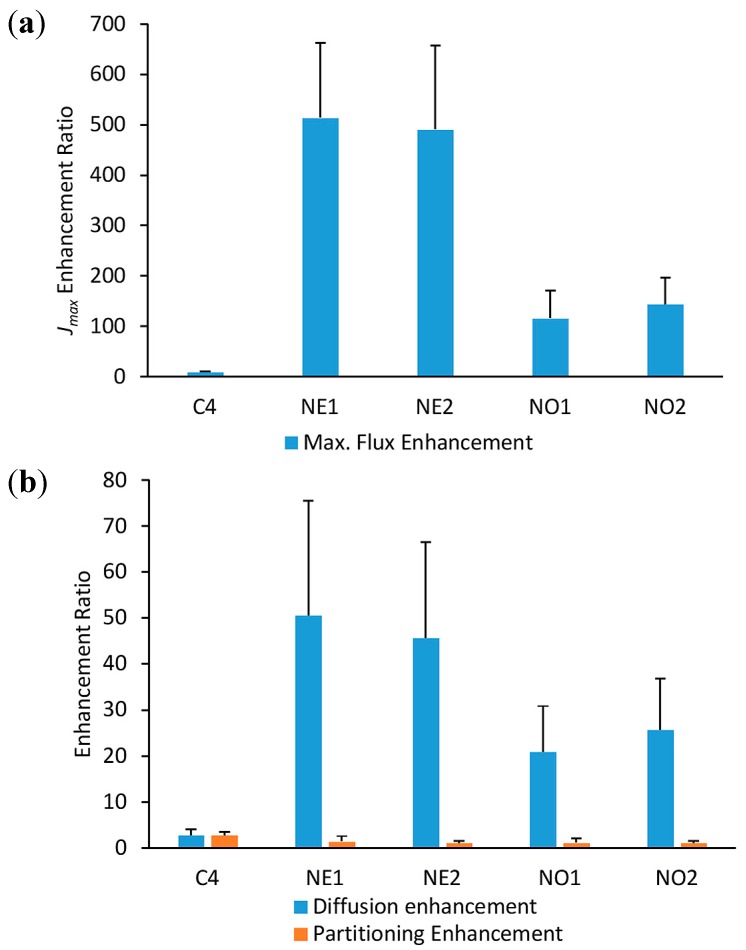
Minoxidil skin permeation enhancement ratios for the 60% ethanol/water formulation (C4) and the four nanoemulsions containing eucalyptol (NE1, NE2) or oleic acid (NO1, NO2), compared to the mean values for the three aqueous control solutions (C1, C2, C3). (**a**) *J_max_* and (**b**) derived diffusivity (D*) and formulation–stratum corneum partition coefficient, *K_f-sc_*.

To explore the different mechanisms of enhancement in the nanoemulsions, the enhancement ratios of maximum flux, diffusivity, and SC–formulation partition coefficient compared to the mean values for the controls (C1–C3) were plotted in Figure 2. It is evident that *J_max_* for minoxidil is greatly enhanced in the nanoemulsion formulations, while the enhancement with the ethanol/water solution is significantly less (Figure 2a). A similar pattern is seen for diffusivity D*, whereas the effect on partitioning from the formulation into the stratum corneum is small (Figure 2b). It is also evident from Table 3 that *J_max_* was independent of minoxidil solubility in the vehicle, *S_f_*, for all the control vehicles, as would be expected for an inert vehicle that had no effect on the skin [21,23]. This was not the case for the vehicles containing ethanol and penetration enhancers.

### 3.3. Minoxidil Retention in the Stratum Corneum, Hair Follicles, and Skin Layers

**Figure 3:**
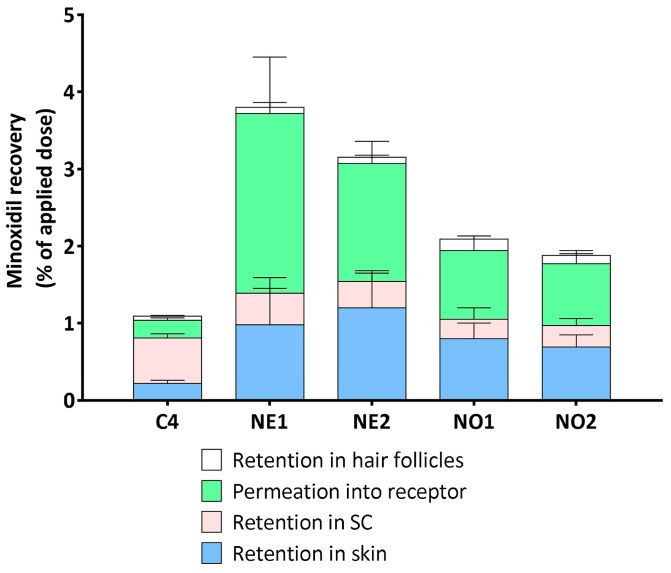
Minoxidil retained in the stratum corneum (SC), hair follicles, and skin layers and permeated through skin. (mean ± SD, *n* = 4).

**Figure 4:**
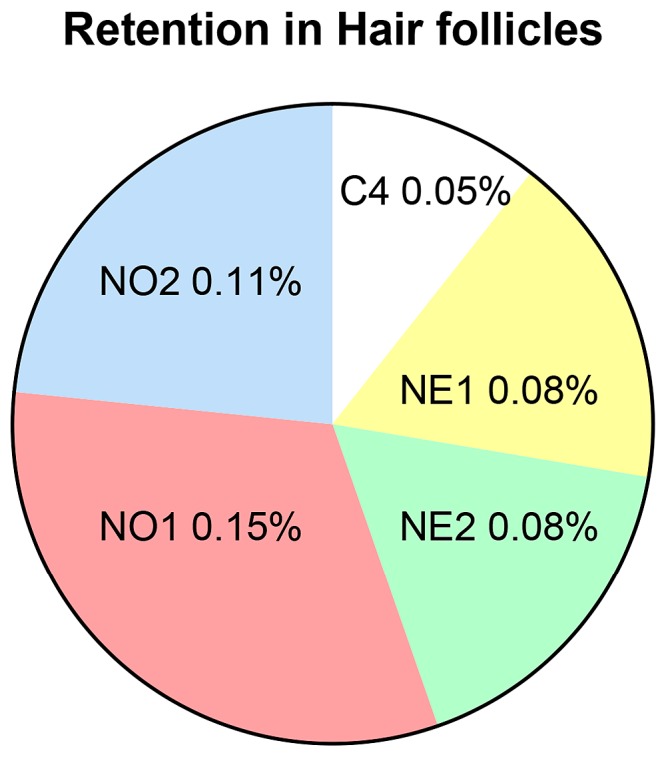
Minoxidil retained in hair follicles (as % of applied dose) from 60% ethanol solution and nanoemulsions. Actual minoxidil delivery ranged from 7.5 µg/cm^2^ (C4) to 22.5 µg/cm^2^ (NO1). *p* < 0.05 for NO1 (oleic acid nanoemulsion) versus 60% ethanol solution (C4); other comparisons not significant.

Minoxidil retention in the various skin compartments (SC, hair follicles, and remaining skin layers), as well as that permeated through skin into the receptor solution, is shown in Figure 3. As there was an insignificant amount of minoxidil retained in the skin after topical application in the three control vehicles C1–C3, we compared only control vehicle C4 (60% ethanol/water) to the nanoemulsions in this section. The greatest minoxidil retention in the SC was from the 60% ethanol/water (C4) control solution (88.0 ± 3.5 µg/cm^2^), followed by the nanoemulsions (37.0 ± 11.0–62.0 ± 15.0). The amounts of minoxidil retained in the deeper skin after 24 h from nanoemulsion formulations were significantly greater than that from control C4 (*p* < 0.05) and were in the order NE2 > NE1 > NO1 > NO2 (Figure 3). The minoxidil recovered from the hair follicles by follicular casting is shown separately in Figure 4, as a percentage of the total applied dose. Follicular delivery was greatest for the nanoemulsions, with the NO greater than the NE. There was a significant difference between follicular delivery from the 60% ethanol solution compared to that from the oleic acid nanoemulsion NO1 (*p* < 0.05; 1-way ANOVA with *post hoc* comparison by Tukey’s test), but other comparisons were not statistically significant. The actual amounts of minoxidil retained in the follicles ranged from 7.5 µg/cm^2^ for the 60% ethanol solution (C4) to 22.5 µg/cm^2^ for the oleic acid nanoemulsion (NO1).

## 4. Discussion

We have shown significant enhancement of skin permeation when minoxidil was applied in nanoemulsion formulations containing OA and EU. This is consistent with previous reports of enhanced delivery of both hydrophilic and lipophilic drugs from nano- and microemulsions containing chemical penetration enhancers [24]. Recently, Roque et al. [25] developed poly(lactic-*co*-glycolic acid) (PLGA) nanoparticles loaded with finasteride for treatment of androgenetic alopecia. Their nanoparticle formulations (lotion, shampoo) and a finasteride solution delivered between 300 and 1000 μg/cm^2^ of finasteride to excised human skin over 24 h, comparable to the maximum delivery of minoxidil seen with our eucalyptol nanoemulsions (350 μg/cm^2^). However, these workers did not assess the delivery and retention of finasteride into the skin or follicles. We have also previously demonstrated that these nanoemulsion formulations gave increased skin delivery of caffeine and naproxen. Three separate mechanisms with the potential to act synergistically may contribute to this effect: (i) enhanced solubility of the drug in the applied nanoemulsions; (ii) increased uptake of the vehicle carrying the drug into the SC; and (iii) a direct effect on the SC membrane properties, such as increased fluidization of the SC lipids. Of particular interest in this study was the determination of delivery via both the SC and the appendages, particularly given the relevance of follicular delivery for minoxidil, a compound that acts on the hair follicles. Grice et al. [18] previously reported that the deposition of minoxidil in appendages was of a similar magnitude to SC penetration following topical application in simple vehicles, thus demonstrating the importance of the follicular route.

The solubility of minoxidil was substantially increased by the nanoemulsion formulations containing a combination of oil phase containing penetration enhancers, surfactant, and cosurfactant, and aqueous phase compared to all solvent mixture control solutions (for example, four- to eightfold greater than the control mixture (C4) containing aqueous ethanol). This is consistent with literature showing that terpene-based formulations provide effective solubilization of moderately lipophilic compounds [26]. We also showed that the increased solubility in the nanoemulsion formulations correlated directly with increased solubility of minoxidil in the stratum corneum, although this was less pronounced when compared with the aqueous ethanol control (1.7- to 3.3-fold). Of the three permeation enhancement mechanisms noted for caffeine and naproxen in our previous study of these nanoemulsions [17], it is clear that for minoxidil the mechanisms of increased solubility in the formulation and vehicle uptake into the SC also apply.

In this current work, we found that there was minimal minoxidil penetration for all formulations up to 4 h, but after 4 h, penetration from the nanoemulsions and aqueous ethanol control increased (Figure 1). Grice et al. [18] also reported a delayed response for minoxidil from solvent mixture vehicles, which was explained in terms of delayed uptake of propylene glycol altering the membrane properties to enhance minoxidil permeation, although the delay was considerably longer in that case. In this work, we found that very little minoxidil permeated though the skin into the receptor fluid by 8 h. Similar results were obtained by Mura et al., who showed only small amounts reaching the epidermis after 8 h [27]. In the present study, we used ethanol as a co-surfactant. As a result of the evaporation of ethanol, the viscosity of the formulations could increase, thereby reducing the solubility of minoxidil in the skin, as well as its thermodynamic activity in the vehicle, leading to delayed skin permeation [28,29]. 

To further explore the mechanisms of enhanced skin permeation, we determined *J_max_*, the maximum (or saturated) flux, as it is well recognized that *J_max_* is independent of the vehicle, but depends solely on the thermodynamic activity of the solute in the vehicle, provided the vehicle or solute does not alter the membrane properties [21,23,30]. It is evident that the control vehicles all had similarly low minoxidil *J_max_* values, indicating that the control vehicles, with a range of minoxidil solubilities, did not affect skin permeation. This is consistent with the report of Twist and Zatz [31], who applied methyl paraben with a range of solubilities in different vehicles to synthetic membranes and showed the same methyl paraben flux in each case, regardless of its vehicle solubility. In contrast, we found that the *J_max_* values for the nanoemulsions were much greater than for controls (Figure 2a). This is consistent with the notion that they have enhanced skin permeability by altering the SC membrane properties. The nanoemulsions were also associated with excellent SC solubility (Table 3). One of the mechanisms by which enhancers such as eucalyptol and oleic acid act is known to involve SC lipid dissolution [32]. In the case of the nanoemulsions used in this study, they appear to be promoting minoxidil penetration by the third mechanism, namely, modification of the SC properties.

An important mechanistic outcome of this work is the strong dependence of *J_max_* on diffusivity, D*, for the enhancer formulations (Figure 2). Overall, diffusivity will be independent from solute lipophilicity if the vehicles do not affect the SC [23,33]. Nonetheless, different effects may be seen when the vehicle affects the SC, as previously seen for hydrophilic phenols which exhibited more pronounced penetration enhancement relative to more lipophilic phenols applied in isopropyl myristate (IPM) [23]. IPM has a high affinity for skin and can disrupt the SC intercellular lipid matrix [23] and so may be expected to also affect minoxidil (log *P* 1.24), consistent with the diffusivity enhancement by nanoemulsions seen here.

Oleic acid and eucalyptol are well-established penetration enhancers. A unique feature of OA is its *cis* double bond at *C_9_* which causes a kink in the alkyl chain and disturbs the SC lipid bilayers [34]. EU also acts by disrupting the SC lipid organization and increasing fluidization which leads to increased drug diffusivity [35]. Obata et al. [36] claimed that ethanol causes greater enhancement in combination with hydrophilic terpenes such as eucalyptol, compared to mixtures containing lipophilic terpenes [37,38]. Here, the EU nanoemulsions, also containing ethanol, caused the greatest penetration of minoxidil. This is also consistent with the enhanced permeation of curcumin from EU-containing microemulsions reported by Liu et al. (2010), who proposed that eucalyptol enters the skin in monomer form and increases drug solubility in the skin, increases partitioning of drugs into the skin, and creates high drug concentrations in the upper layers of the skin [39]. Our results showing the greatest SC solubility with EU nanoemulsions are consistent with this mechanism.

The OA nanoemulsions showed greater minoxidil retention in the hair follicles than did the EU formulations. This enhanced follicular penetration is likely associated with the minoxidil solubility in the nanoemulsions and the compatibility of the nanoemulsions with the lipophilic sebum environment of the sebum [40]. In contrast, EU nanoemulsions showed greater retention of minoxidil in the SC. These results suggest that the mechanism of drug transport across skin depends on the composition of the formulation and the solute used. 

## 5. Conclusions

In conclusion, the nanoemulsion systems in this study significantly enhanced the permeation of minoxidil through full-thickness human skin. Both nanoemulsions increased the diffusivity of minoxidil and its solubility in the stratum corneum. While the mechanism of penetration enhancement has been suggested to involve increases in both partitioning and diffusivity, depending on the deliverable and the vehicle, in this study, the influence of diffusivity far outweighed that of increased partitioning. Furthermore, the enhanced minoxidil deposition seen in hair follicles in this study suggests that nanoemulsions may be suitable vehicles to deliver substances into appendages for therapeutic purposes. Further formulation optimization is likely to result in greater drug delivery by this route. Work is currently in progress to characterize the skin irritancy potential of these nanoemulsion formulations containing penetration enhancers.

## Figures and Tables

**Table 1:** Compositions (% *w*/*w*) of control solutions (C1–C4) and nanoemulsion formulations with penetration enhancers eucalyptol (NE1 and NE2) and oleic acid (NO1 and NO2). The concentration of minoxidil in aqueous controls and nanoemulsions was 2% *w*/*w*, except in C1, which was a saturated solution.

Formulation	Water	Ethanol	PEG-6000	Volpo-N10	Eucalyptol	Oleic Acid
C1	60		35			
C2	50		50			
C3	25		75			
C4	40	60				
NE1	30.97	26.55		26.55	15.93	
NE2	36.59	24.39		24.39	14.63	
NO1	30.97	26.55		26.55		15.93
NO2	36.59	24.39		24.39		14.63

**Table 2:** Physical and chemical characterization of the various nanoemulsion formulations defined in Table 1.

PROPERTY	NE1	NE2	NO1	NO2
Appearance	Clear	Clear	Clear	Clear
Viscosity (cp)	13.7 ± 4.5	15.1 ± 4.0	23.0 ± 4.7	28.3 ± 4.5
Conductivity (µS)	87.5 ± 2.2	91.3 ± 3.9	80.8 ± 8.2	84.5 ± 10.1
Refractive Index	1.38	1.37	1.38	1.37
Droplet size (nm) (emulsion only)	29.6 ± 3.1	19.5 ± 1.3	8.0 ± 0.5	12.4 ± 0.1

**Table 3:** Experimental data for minoxidil in different nanoemulsions and control vehicles. (Mean ± SD). The nanoemulsions containing penetration enhancers eucalyptol (NE1 and NE2) and oleic acid (NO1 and NO2), and the control solutions (C1–C4), are defined in Table 1.

Formulation	*J_SS_*	*J_max_*	D*	*S_f_*	*S_SC_*
	(µg/cm^2^/h)	(µg/cm^2^/h)	(cm/h*1E4)	(mg/mL)	(mg/mL)
C1	0.4 ± 0.1	0.4 ± 0.1	0.3 ± 0.1	15.5 ± 2.3	13.2 ± 0.4
C2	0.2 ± 0.1	0.4 ± 0.1	0.3 ± 0.1	39.0 ± 0.9	12.3 ± 4.3
C3	0.1 ± 0.0	0.4 ± 0.2	0.2 ± 0.2	73.0 ± 1.2	15.5 ± 0.4
C4	1.9 ± 0.1	3.3 ± 0.2	0.8 ± 0.0	34.7 ± 2.7	44.3 ± 4.6
NE1	18.1 ± 2.3	188.4 ± 23.4	13.8 ± 0.1	207.8 ± 1.6	136.1 ± 16.9
NE2	12.7 ± 1.9	179.9 ± 27.1	12.5 ± 1.8	283.4 ± 2.3	143.9 ± 13.4
NO1	6.2 ± 1.9	42.6 ± 12.9	5.7 ± 2.4	137.3 ± 1.1	74.2 ± 8.0
NO2	6.9 ± 1.2	52.8 ± 9.0	7.0 ± 1.2	152.1 ± 1.3	75.1 ± 5.7
